# Supplementary material for: MetaQTL: a package of new computational methods for the meta-analysis of QTL mapping experiments
Source: BMC Bioinformatics. 2007 Feb 8;8:49. doi: 10.1186/1471-2105-8-49 (PMC1808479; doi:10.1186/1471-2105-8-49)
Supplement: Additional File 7 — MetaQTL Package : jar file and tutorial. This Zip archive contains both the MetaQTL JAR file and the files of the tutorial. [file 1471-2105-8-49-S7.zip › org.inra.metaqtl/doc/org/metaqtl/class-use/MetaQtlModel.html]

Uses of Class org.metaqtl.MetaQtlModel


|  |  |  |
| --- | --- | --- |
| **Overview** \| **Package** \| **Class** \| **Use** \| **Tree** \| **Deprecated** \| **Index** \| **Help** | |  |
| PREV   NEXT | **FRAMES**    **NO FRAMES**     **All Classes** |  |


---


## **Uses of Class org.metaqtl.MetaQtlModel**

| Packages that use MetaQtlModel | |
| --- | --- |
| **org.metaqtl** |  |
| **org.metaqtl.factory** |  |
| **org.metaqtl.main** |  |

Uses of MetaQtlModel in org.metaqtl

| Methods in org.metaqtl that return MetaQtlModel | |
| --- | --- |
| `MetaQtlModel` | `MetaQtlAnalysis.getBestModel(int criterion)`             This methods returns the best models for each criterion defined to select the optimal number of clusters per chromosome per trait. |

Uses of MetaQtlModel in org.metaqtl.factory

| Methods in org.metaqtl.factory that return MetaQtlModel | |
| --- | --- |
| `static MetaQtlModel` | `MetaQtlModelFactory.read(java.io.Reader reader)`             This method reads a file formated as follows : Chromosome \t Trait \t Model |

| Methods in org.metaqtl.factory with parameters of type MetaQtlModel | |
| --- | --- |
| `static void` | `MetaQtlModelFactory.write(MetaQtlModel model, java.io.OutputStream stream)` |
| `static void` | `MetaQtlModelFactory.write(MetaQtlModel model, java.io.Writer writer)` |

Uses of MetaQtlModel in org.metaqtl.main

| Methods in org.metaqtl.main that return MetaQtlModel | |
| --- | --- |
| `protected static MetaQtlModel` | `MetaMain.getCluster(java.lang.String cluFile)` |

---


|  |  |  |
| --- | --- | --- |
| **Overview** \| **Package** \| **Class** \| **Use** \| **Tree** \| **Deprecated** \| **Index** \| **Help** | |  |
| PREV   NEXT | **FRAMES**    **NO FRAMES**     **All Classes** |  |


---
